# Identification of an *ABCC8* variant in a kindred with transient diazoxide responsive hyperinsulinism

**DOI:** 10.1530/EDM-24-0106

**Published:** 2025-07-03

**Authors:** Ryan L Smith, Stephen I Stone

**Affiliations:** Deparment of Pediatrics, Washington University in St. Louis School of Medicine, St. Louis, Missouri, USA

**Keywords:** congenital hyperinsulinism, genetics, diazoxide

## Abstract

**Summary:**

Congenital hyperinsulinism is a rare disorder characterized by hypoglycemia and inappropriately elevated insulin levels. The genetics of congenital hyperinsulinism is complex, with the most common cause being pathogenic variants in the ATP-sensitive potassium channel. Depending on the parent of origin, patients may present with focal or diffuse hyperinsulinism. Typically, patients with focal hyperinsulinism are non-responsive to diazoxide and likely progress to surgical therapy. However, there can be exceptions to these rules. We evaluated two siblings with congenital hyperinsulinism. Genetic testing identified a paternally inherited variant in *ABCC8*. One sibling had significant neonatal hypoglycemia requiring diazoxide for several years before weaning off daily diazoxide, whereas the second sibling experienced transitional hypoglycemia in the neonatal period but only requires diazoxide therapy during periods of intercurrent illness. This case highlights the importance of genetic testing for congenital hyperinsulinism.

**Learning points:**

- The most common genetic cause of hyperinsulinism is gain-of-function variants in *ABCC8* and *KCNJ11*, which make up the ATP-sensitive potassium channel (K_ATP_).
- First-degree relatives of affected individuals should be considered for genetic testing.
- Parent-of-origin testing should be done to determine if the patient is likely to have focal or diffuse hyperinsulinism.
- Diazoxide is helpful for many patients with diffuse hyperinsulinism, and some patients with focal hyperinsulinism.

## Background

Hyperinsulinism (HI) in infants is characterized by prolonged and recurrent episodes of hypoglycemia. The diagnosis is made when a child has a blood glucose level less than 50 mg/dL and an inappropriately elevated insulin level (1). As there is a high degree of morbidity associated with HI, expert care with an experienced endocrinology team is preferred (2).

Most cases of HI are transient. A major risk factor for hyperinsulinism is gestational diabetes or a mother with preexisting diabetes. Additional risk factors include maternal hypertension, being born large for gestational age, small for gestational age, traumatic delivery, and birth asphyxia. These cases often respond well to diazoxide and often resolve before 6 months of age (3, 4). A subset of patients with HI will go on to experience prolonged hypoglycemia. These patients are known to have congenital hyperinsulinism (CHI). These cases can be subdivided into patients who are diazoxide responsive and diazoxide non-responsive. An additional distinction can be made based on whether the HI is focal (localized to a specific portion of the pancreas) or diffuse (localized throughout the entire pancreas). Oftentimes, patients with focal HI are diazoxide non-responsive. Diazoxide non-responsive patients may require partial or subtotal pancreatectomy (5).

There are interesting genetics related to CHI. The most common genetic variants responsible for CHI are *ABCC8* (SUR1) and *KCNJ11* (Kir6.2), which form the ATP-sensitive potassium (K_ATP_) channel of the pancreatic β-cell. Loss-of-function variants in either of these genes result in closure of the K_ATP_ channel. This depolarizes the β-cell membrane, leading to inappropriate and unregulated release of insulin regardless of glucose status (5). Imprinting occurs on both the *ABCC8* and *KCNJ11* loci; as such, when paternally inherited variants often result in focal HI, and maternally inherited variants result in diffuse HI (6).

Due to the particular genetics of CHI, establishing a genetic diagnosis as well as parent of origin is extremely important for long-term management (5). As this is a growing field, we continue to identify new cases of CHI with novel variants. Here, we present two siblings with monoallelic, paternally inherited variants in *ABCC8* who present with varying degrees of diazoxide-responsive CHI. Such cases widen our collective understanding of the spectrum of CHI and add to our knowledge of the spectrum of *ABCC8*-related HI.

## Case presentation

### Case 1 (proband)

#### Presentation

A male infant was born to a 30-year-old G1P0 mother at 38 and 2/7 week gestational age via cesarean section due to breech presentation. The birth weight was 3,320 g and length was 49.5 cm. Pregnancy was notable for a failed 1 h screening glucose tolerance test; however, his mother passed the 3 h oral glucose tolerance test (OGTT). There was a maternal history of depression and anxiety, not treated with medication. In addition, the patient’s mother had a history of a bicornuate uterus.

APGAR scores were 8 and 9. However, shortly after birth, the patient developed tachypnea. His initial blood glucose was 30 mg/dL via point-of-care measurement. He was brought to the neonatal intensive care unit (NICU) for further evaluation. Physical exam was unremarkable except for bilateral undescended testes.

#### Investigation

Critical hypoglycemia labs drawn at 12 h of life were significant for hypoglycemia (glucose: 27 mg/dL), an inappropriately elevated insulin (11.0 µIU/dL) level, and an inappropriately low cortisol (5.1 μg/dL). Urine ketones were negative. He required increasing titration of his glucose infusion rate (GIR) initially from 5.5 mg/kg/min up to 20.8 mg/kg/min. This was consistent with a diagnosis of hyperinsulinism.

In response to the low cortisol level, the patient underwent a low-dose (1 μg) ACTH stimulation test. The patient failed the test, with cortisol levels of <1.5 μg/dL, 3.2 μg/dL, and 4.6 μg/dL at 0, 20, and 30 min respectively. Baseline ACTH was inappropriately normal at 23.0 pg/mL. Given this result, he was briefly started on physiologic hydrocortisone replacement before discharge from the NICU.

Genetic testing was done to determine if there was a genetic contribution to the patient’s hyperinsulinism. Sanger sequencing (Athena Diagnostics) identified a variant of unknown significance (VUS) in the gene *ABCC8* (c.1252T>C; Cys418Arg) (Table 1). Cascade testing was done in the proband’s parents, identifying that the patient’s father also carried the same *ABCC8* variant; however, he was clinically unaffected with no history of hypoglycemia.

**Table 1:** *In silico* analysis of *ABCC8* variant in exon 8.

HGNC Symbol	*ABCC8*
GenBank transcript ID	NM_000352
DNA change	c.1252T>C
Genomic DNA change	g.28307T>C
Alteration location	chr11:g.17448596A>G
Amino acid change	p.Cys418Arg
gnomAD frequency	0.0009249
SIFT	0.044
PolyPhen2	0.35
CADD-PHRED	27.2
REVEL	0.66

In addition to hyperinsulinism testing, the proband underwent FISH for SRY due to his undescended testes. A chromosomal microarray was also performed. This demonstrated a maternally inherited duplication in 3p26.3 (D3S4559), which has been associated with neurocognitive delays (7). In addition, the proband harbored a *de novo* 100 kb deletion in 15q15.3, which has been reported in cases of sensorineural deafness (8).

#### Treatment

The patient was treated with IV fluids containing 25% dextrose. His GIR reached up to 21 mg/kg/min. Given his low cortisol levels, he was started on stress doses of hydrocortisone. After confirming the diagnosis of hyperinsulinism, the patient was started on diazoxide. He was titrated to a dose of 10 mg/kg/day divided three times daily. Over the next several days, he was able to wean off IV dextrose and transition to full enteral feeds without hypoglycemia. Before discharge from the NICU, he tolerated a 6 h safety fast without hypoglycemia.

### Case 2

#### Presentation

The proband’s mother was evaluated in the prenatal period with endocrinology and neonatology in the fetal care clinic. Amniocentesis with genetic testing for *ABCC8* was recommended; however, as the variant was classified as a VUS, insurance coverage was denied. Plans were made to evaluate the infant for hypoglycemia shortly after birth.

The proband’s sister was born via scheduled repeat cesarean section at 38 3/7 weeks gestational age. Her initial blood glucose was 40 mg/dL, and she was transferred to the NICU for monitoring. She was started on IV fluids with 10% dextrose. However, her blood glucoses levels remained stable, and she was weaned off IV fluids by day 3 of life. She tolerated a 6 h safety fast before discharge. As she never developed significant hypoglycemia, a critical sample (including insulin and cortisol) was not obtained, and she was never started on diazoxide.

#### Investigation

Given the diagnosis of her brother, targeted testing for the *ABCC8* variant was performed. She was found to have the same paternally inherited variant in *ABCC8*. In addition, she underwent a chromosomal microarray. This demonstrated the same maternally inherited duplication in 3p26.3 (D3S4559) as present in case 1. In addition, she carried a *de novo* 612 kb duplication on 2p15.

#### Treatment

Considering the genetic findings and transitional hypoglycemia in the newborn period, she was presumed to have mild congenital hyperinsulinism. As her brother has been diazoxide responsive, she was given a prescription for diazoxide to use in case of intercurrent illnesses. She also has a home glucometer and emergency glucagon.

## Outcome and follow-up

Both patients have followed up with our clinic for several years (7 and 9 years old for case 2 and case 1 respectively). Case 1 underwent a repeat low-dose ACTH test at 5 weeks old, after transition to dexamethasone. This test demonstrated cortisol levels of 13.8 μg/dL, 23.0 μg/dL, and 25.9 μg/dL at 0, 20, and 30 min respectively. Baseline ACTH was 53.6 pg/mL. We concluded that he likely had hypoglycemia-associated autonomic failure due to recurrent hypoglycemia. However, this had resolved since starting diazoxide. Given these results, the proband was weaned off hydrocortisone.

Case 1 continued daily diazoxide but had significantly outgrown his dose (0.7 mg/kg/day). At age 9, his parents weaned off the diazoxide, and he was able to maintain normal glucose levels. Case 2 was never started on daily diazoxide. However, both patients have experienced symptomatic hypoglycemia during periods of intercurrent gastrointestinal illness. On these occasions, they require IV fluids with dextrose. They have responded well to larger doses of diazoxide in the 10–15 mg/kg/day range. Therefore, they have sick day instructions to give diazoxide as needed and carry glucagon in case of severe hypoglycemia.

Case 1 has an individualized education plan and receives speech therapy. He also carries a diagnosis of attention-deficit or hyperactivity disorder. At 8 years old, he underwent surgery to repair his undescended testes without complication. Case 2 has done well in school and is neurodevelopmentally normal.

## Discussion

The cases presented in this manuscript highlight the unique genetics of CHI. Notably, both siblings harbor the same paternally inherited variant in *ABCC8*. However, case 1 is more severely affected when compared to case 2. Most cases of CHI due to paternally inherited variants in the K_ATP_ channel result in focal hyperinsulinism (9). Therefore, we hypothesize that the variant in *ABCC8* is autosomal dominant with variable expressivity. This is likely due to the extent of involvement of each subject’s endocrine pancreas. Review of this variant in gnomAD v.4.1 demonstrates a minor allele frequency of 0.0009249. There is a maximum population frequency of 0.001118 (1,319/1,180,038, with 1 homozygote) in the European non-Finnish cohort (10). The specific *ABCC8*:c.1252T>C variant has been reported previously in patients with HI (11, 12).

Most cases of focal hyperinsulinism are refractory to diazoxide (5). However, both cases highlight the importance of doing a diazoxide trial. Despite the paternally inherited variant in *ABCC8*, both subjects were diazoxide responsive. Notably, this specific variant of *ABCC8* has been found to be diazoxide responsive (13). Thus, patients with the same or similar variants may benefit from diazoxide therapy. Our team considered performing a PET imaging with 18-F-L 3,4-dihydroxyphenylalanine ([18F]-FDOPA) in case 1, however, we declined to do so as he was diazoxide responsive (14). In addition, this case delineates the spectrum of hyperinsulinism, as both cases are exquisitely diazoxide responsive, now only needing to take diazoxide during intercurrent illnesses.

**Figure 1:**
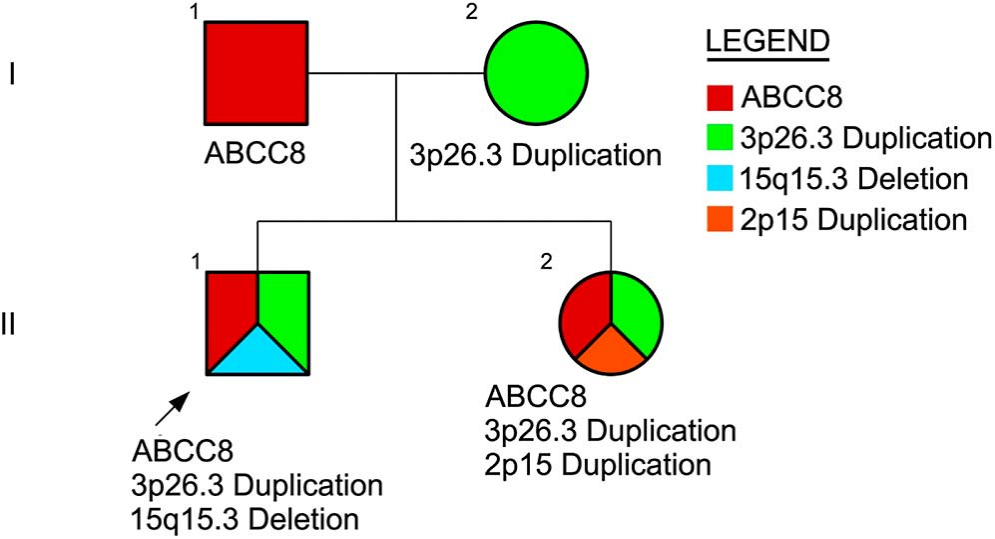
Pedigree of the kindred described, harboring the pathogenic *ABCC8* variant. II-1 is the proband with congenital hyperinsulinism. He remained on diazoxide until age 9. II-2 had transitional hypoglycemia as a neonate and currently requires diazoxide during periods of intercurrent illness. The father (I-1) is an asymptomatic carrier.

A confounding aspect of these cases is the result of their chromosomal microarray. As noted in Fig. 1, both cases 1 and 2 harbor maternally inherited duplication of 3p26.3. Interestingly, case 1 also harbors a *de novo* deletion on chromosome 15q16.3 (100 kb), and case 2 harbors a *de novo* duplication on chromosome 2p15 (612 kb). There is a chance that these additional deletions and duplications may be contributing to or modifying the clinical presentations of these patients.

Strengths of this study include careful genotyping of the family to establish a genetic basis of their CHI. Sanger sequencing of the proband, and subsequently the parents and the sibling, allowed for early diagnosis and phasing of the variant. Both cases have been followed with our team for several years, contributing significantly to our understanding of their symptoms.

The results of our study are limited, as this is a single kindred. However, we hope that publishing these findings will contribute to the knowledge of the spectrum of CHI cases related to *ABCC8*. This information may support upgrading this variant to likely pathogenic (15). For this family, the mother was denied insurance coverage for amniocentesis, as the *ABCC8* variant was classified as a variant of uncertain significance. This would have aided in pre- and post-natal planning for case 2, potentially averting exposure to hypoglycemia.

## Declaration of interest

The authors declare that there is no conflict of interest that could be perceived as prejudicing the impartiality of the reported research.

## Funding

This research did not receive any specific grant from any funding agency in the public, commercial, or not-for-profit sector. However, SIS is supported by a K08 grant from the National Institutes of Health (NIH) (K08DK124574), and a Faculty Recruitment Scholar Award from the Children’s Discovery Institute at Washington University in St. Louis.

## Patient consent

Written consent has been obtained from each patient after full explanation of the purpose and nature of all procedures used.

## Author contribution statement

RLS and SIS contributed to the conception and design of this manuscript.
